# Readability of Patient-Facing Information of Antibiotics Used in the WHO Short 6-Month and 9-Month All Oral Treatment for Drug-Resistant Tuberculosis

**DOI:** 10.1007/s00408-024-00732-z

**Published:** 2024-07-26

**Authors:** John E. Moore, Beverley C. Millar

**Affiliations:** 1 School of Biomedical Sciences, Ulster University, Cromore Road, Coleraine, Northern Ireland BT52 1SA UK; 2 Laboratory for Disinfection and Pathogen Elimination Studies, Northern Ireland Public Health Laboratory, Belfast City Hospital, Lisburn Road, Belfast, Northern Ireland BT9 7AD UK

**Keywords:** Antibiotics, Antibiotic resistance, Readability, Treatment literacy, Tuberculosis

## Abstract

**Objectives:**

Readability of patient-facing information of oral antibiotics detailed in the WHO all oral short (6 months, 9 months) has not been described to date. The aim of this study was therefore to examine (i) how readable patient-facing TB antibiotic information is compared to readability reference standards and (ii) if there are differences in readability between high-incidence countries versus low-incidence countries.

**Methods:**

Ten antibiotics, including bedaquiline, clofazimine, ethambutol, ethionamide, isoniazid, levofloxacin, linezolid, moxifloxacin, pretomanid, pyrazinamide, were investigated. TB antibiotic information sources were examined, consisting of 85 Patient Information Leaflets (PILs) and 40 antibiotic web resouces. Of these 85 PILs, 72 were taken from the National Medicines Regulator from six countries (3 TB high-incidence [Rwanda, Malaysia, South Africa] + 3 TB low-incidence [UK, Ireland, Malta] countries). Readability data was grouped into three categories, including (i) high TB-incidence countries (*n* = 33 information sources), (ii) low TB-incidence countries (*n* = 39 information sources) and (iii) web information (*n* = 53). Readability was calculated using Readable software, to obtain four readability scores [(i) Flesch Reading Ease (FRE), (ii) Flesch-Kincaid Grade Level (FKGL), (iii) Gunning Fog Index and (iv) SMOG Index], as well as two text metrics [words/sentence, syllables/word].

**Results:**

Mean readability scores of patient-facing TB antibiotic information for FRE and FKGL, were 47.4 ± 12.6 (sd) (target ≥ 60) and 9.2 ± 2.0 (target ≤ 8.0), respectively. There was no significant difference in readability between low incidence countries and web resources, but there was significantly poorer readability associated with PILs from high incidence countries versus low incidence countries (FRE; *p* = 0.0056: FKGL; *p* = 0.0095).

**Conclusions:**

Readability of TB antibiotic PILs is poor. Improving readability of PILs should be an important objective when preparing patient-facing written materials, thereby improving patient health/treatment literacy.

**Supplementary Information:**

The online version contains supplementary material available at 10.1007/s00408-024-00732-z.

## Introduction

Tuberculosis (TB) continues to be the most significant global health crisis, with approximately more than 1 billion people having died with the disease over the past two centuries [1, 2]. Worldwide, TB is the second leading infectious killer after COVID-19 (above HIV and AIDS), where it is estimated that 1.3 million people died from TB in 2022 (including 167 000 people with HIV) [2]. In 2022, an estimated 10.6 million people fell ill with tuberculosis (TB) worldwide, including 5.8 million men, 3.5 million women and 1.3 million children [2]. More recently, antibiotic-resistant forms of the disease have emerged and in 2022, where 410,000 (3.9% of new TB cases) were some form of antibiotic-resistant TB [2] [See Supplementary File 1]. The various forms of antibiotic-resistant TB often consume > 50% of national TB budgets despite comprising < 5–10% of the total TB case-load [3]. Antibiotic-resistant TB can be defined on the basis of resistance to various antibiotics or combinations of antibiotics, as detailed in Table 1 [4]. Two short, all-oral antibiotic regimens for multidrug-resistant TB have been proposed by the WHO, which have now been adopted [4]. The BPaLM regimen (6 Bdq-Pa-Lzd-Mfx1) is employed in patients with MDR/RR-TB where fluoroquinolone susceptibility is presumed or documented [4]. This 6-month all-oral treatment regimen comprises bedaquiline, pretomanid, linezolid and moxifloxacin, where it is possible to omit moxifloxacin and continue with the BPaL regimen for MDR/RR-TB patients with confirmed fluoroquinolone resistance [4]. The slightly longer 9-month all-oral regimen (4–6 Bdq(6m)-Lfx/Mfx-Cfz-Z-E-Hh-Eto or Lzd(2m)/5 Lfx/Mfx-Cfz-Z-E) is employed in patients with MDR/RR-TB and in whom resistance to fluoroquinolones has been excluded [4]. This 9-month all-oral regimen comprises bedaquiline (used for 6 months), in combination with levofloxacin/moxifloxacin, ethionamide, ethambutol, isoniazid (high dose), pyrazinamide and clofazimine (for 4 months, with the possibility of extending to 6 months if the patient remains sputum smear positive at the end of 4 months), followed by treatment with levofloxacin/moxifloxacin, clofazimine, ethambutol and pyrazinamide (for 5 months), where ethionamide can be replaced by 2 months of linezolid [4].

**Table 1:** World Health Organization definitions of seven forms of antibiotic-resistant TB

Abbreviation	Description
Drug-resistant TB (DR-TB):	TB disease caused by a strain of *Mycobacterium tuberculosis* complex that is resistant to any TB medicine
Extensively drug-resistant TB (XDR-TB):	TB disease caused by a strain of *M. tuberculosis* complex that is resistant to rifampicin (and may also be resistant to isoniazid), and that is also resistant to at least one fluoroquinolone (levofloxacin or moxifloxacin) and to at least one other “Group A” drug (bedaquiline or linezolid)
MDR/RR-TB:	Refers to either multidrug-resistant TB (MDR-TB) or rifampicin-resistant TB (RR-TB)
Multidrug-resistant TB (MDR-TB):	TB disease caused by a strain of *M. tuberculosis* complex that is resistant to rifampicin and isoniazid
Pre-extensively drug-resistant TB (pre-XDR-TB):	TB disease caused by a strain of M. tuberculosis complex that is resistant to rifampicin (and may also be resistant to isoniazid), and that is also resistant to at least one fluoroquinolone (either levofloxacin or moxifloxacin)
Rifampicin-resistant TB (RR-TB):	TB disease caused by a strain of *M. tuberculosis* complex that is resistant to rifampicin. These strains may be susceptible or resistant to isoniazid (i.e. MDR-TB), or resistant to other first-line or second-line TB medicines
Rifampicin-susceptible, isoniazid-resistant TB (Hr-TB):	TB disease caused by a strain of *M. tuberculosis* complex that is resistant to isoniazid but susceptible to rifampicin

Definitions taken from WHO operational handbook on tuberculosis Drug-resistant tuberculosis treatment Module 4: Treatment 2022 update (Available at https://www.who.int/publications/i/item/9789240065116)

Employment of an all oral antibiotic regime, no longer requires the employment of iv antibiotics that require administration with the support of a healthcare professional (nurse), within a healthcare facility or at home as an out-patient. Such administration promotes treatment adherence to taking the antibiotics in accordance with the dosing schedule, as stated in the summary of product characteristics (SPC) of each antibiotic. Many DR-TB programmes implement directly observed treatment (DOT) for people on drug-resistant TB treatment, including those on all-oral medications. Even though treatment of DR-TB has evolved to injection-free regimens, treatment delivery has continued to be DOT. DOT puts the responsibility of adherence on the healthcare provider. However, patients feel that facility-based DOT perpetuates stigma, hinders collection and administration of treatment, and inhibits return to daily activities [5]. More recently, community-supported self-administered treatment (SAT) of TB medication has been introduced, where this is a model in which patients are not supervised daily but receive regular support visits in their community [5]. Employment of an all oral regime under SAT largely transfers the responsibility for antibiotic treatment adherence to the patient, as a healthcare professional is longer required to administer iv antibiotics nor are present to perform DOT.

Non-adherence with oral antibiotic regimes has been shown to reduce clinical efficacy and adversely affect treatment [6–8]. Such behaviour can then led to increased antibiotic resistance in the targeted bacterial pathogen, due to suboptimal pharmacokinetics and pharmacodynamics (PK/PD) values, thereby allowing the pathogen to develop eloborate evasive mechanisms to circumvent the action of the antibiotic [9].

To date, there has not been any reports examining the readability of Patient Information Leaflets (PILs), relating to the oral antibiotics employed in the short 6-months and 9-months all oral antibiotic treatment regimes. Another potential factor which may potentially affect patient antibiotic adherence, is the impact of the readability of these antibiotic PILs. PILs are enclosed with prescription drugs by the dispensing pharmacist and these are crucial in providing key information to the patient about antibiotic regimen, dose, administration, side effects and safety precautions. A previous study has indicated the importance of evaluating the readability of PILs attached to medication, as low-quality information provided could potentially lead to increased patient misusing and cause lower adherence to taking antibiotics correctly [10]. The consequence of poor adherence to antibiotics could potentially lead to the development of AMR, due to the presence of suboptimal minimum inhibitory concentrations (MICs) of antibiotics and thus lead to poor health outcomes.

Readability can be assessed through a range of quantitative readability parameters and formulae based on various text metrics such as word count, sentence count and syllables [11]. Some readability formulae commonly used in healthcare include the Flesch-Kincaid Grade Level (FKGL) and the Flesch Reading Ease (FRE) scores [11] (see Supplementary Table 1). To date, there has not been any research conducted that has examined the readability of patient-facing materials including PILs for those antibiotics used in the WHO short all oral (6-month & 9-month) Drug-Resistant TB treatment regimes. If antibiotic patient information which accompanies those antibiotics is poor, then the patients may be less likely to understand how and why they should take their antibiotics, which may result in them not properly or consistently taking their antibiotics as required. A good understanding of medication instruction is therefore vital for an individual to adequately comprehend and follow the recommended intake and dose of medication, in a way to maximise health outcomes and an additional way to minimise potential contributing factors to AMR.

The aim of this study was therefore to examine the readability (Flesch Reading Ease, Flesch-Kincaid Grade Level, Gunning Fog, SMOG scores; text metrics) of PILs of antibiotics (*n* = 10) used in the WHO short all oral (6-month & 9-month) Drug-Resistant TB treatment regimes, from TB high-incidence countries (*n* = 3), low-incidence countries (*n* = 3), as well as respected web resources (*n* = 4), in order to establish:-

(i) how readable patient-facing antibiotic information is compared to readability reference standards,
(ii) if there are differences in readability between high-incidence countries versus non-high incidence countries versus web resources.

## Materials and Methods

**Fig. 1:**
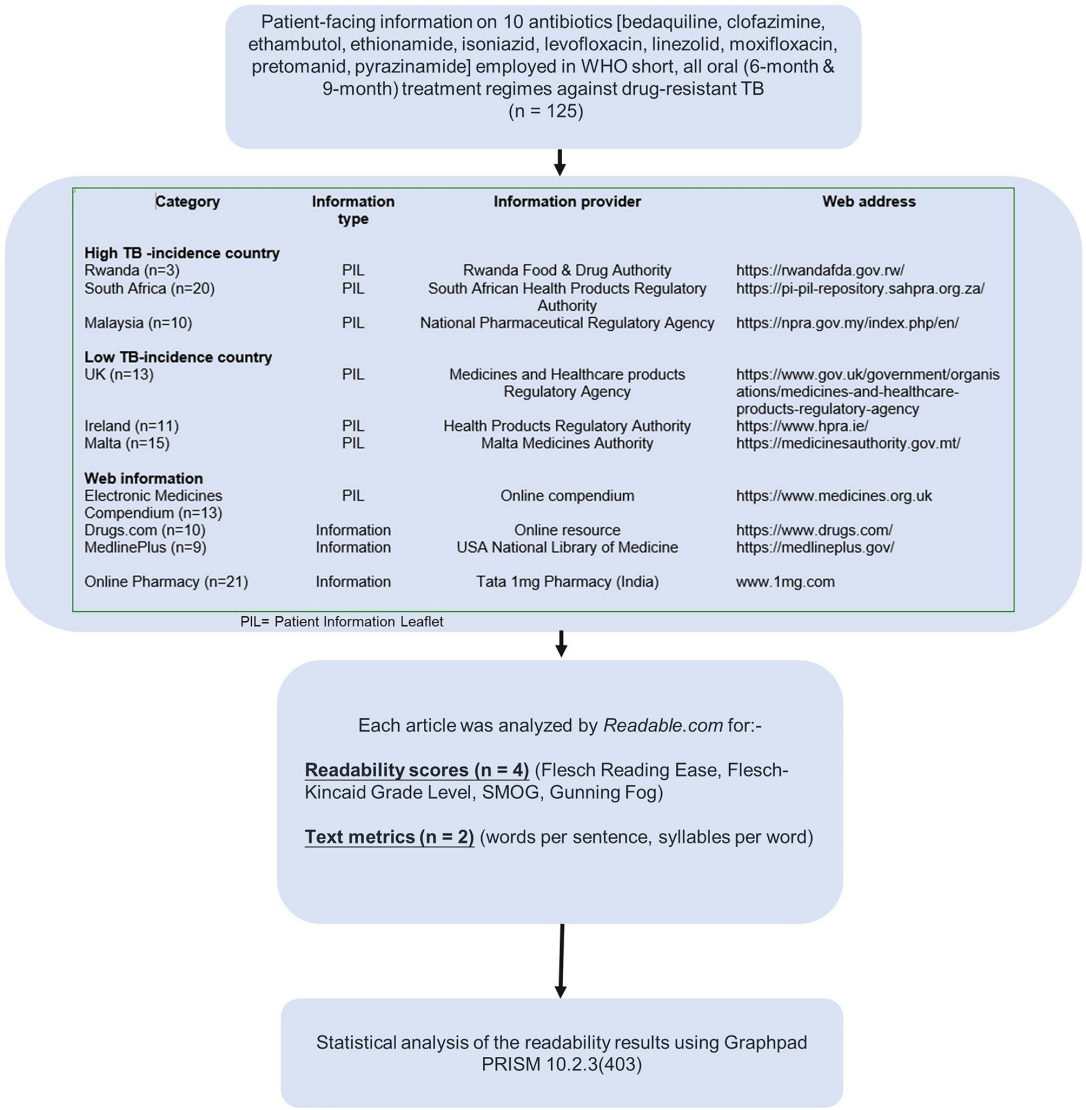
Flow diagram of methodological investigations undertaken in this study and sources of patient-facing information

An overview of the methods employed is shown in Fig. 1.

### Retrieval of Patient Information Leaflets (PILs) of Antibiotics from WHO Short, All Oral (6-Month & 9-Month) Treatment Regimes

Oral antibiotics (*n* = 10), which are employed in the WHO short, all oral (6-month & 9-month) Drug-Resistant TB treatment regimes and used to treat Drug-Resistant (DR) TB, were selected for investigation [4]. These included bedaquiline, clofazimine, ethambutol, ethionamide, isoniazid, levofloxacin, linezolid, moxifloxacin, pretomanid, pyrazinamide.

Patient Information (*n* = 125 sources) aimed at patients and the general public were obtained from publicly and freely available web resources, as detailed in Fig. i1.

### Determination of Readability Scores and Text Metrics

Each PIL in form of a PDF document and each patient information resource, in the form of a URL, was examined using the online subscription-based software, *Readable* (www.readable.com), which was used in accordance with the website’s instructions. All readability analyses were performed on text written in the English language. The software was used to calculate four readability scores, including (i) Flesch Reading Ease, (ii) Flesch-Kincaid Grade Level, (iii) Gunning Fog Index and (iv) SMOG Index, as detailed in Supplementary Table 1. An additional two text metrics were also calculated, including words per sentence and syllables per word. These readability measures were chosen for examination as most readability studies frequently employ these [12, 13]. Readable.com was selected as the preferred online calculator, as it has been previously used in several healthcare readability studies, [12, 13] as well as in a recent study which compared a variety of online readability calculators and concluded that *Readable* was the optimum calculator to use due to its accuracy, user experience and capacity to examine multiple readability parameters from clinical materials [14].

### Statistical Analyses

The readability data obtained underwent statistical analyses using GraphPad PRISM version 10.2.3 (403) (Boston, USA). To determine if the data followed a normal distribution, a normality test was performed on each set of data using the Shapiro-Wilk Test. Dependent on the normality of data distribution, for data that were normally distributed, one-way ANOVA (parametric) was performed to compare the means of normally distributed parameters. Data sets that were not normally distributed, the Kruskal–Wallis (non-parametric) test with Dunn’s Adjusted *p* values was performed. A *p* value of < 0.05 (5%) was considered as statistically significant.

## Results

### Comparison of Readability Scores and Text Metrics of 10 Antibiotics from WHO Short, All Oral (6-Month & 9-Month) Treatment Regimes

A total of 125 antibiotic information sources were examined, consisting of 85 Patient Information Leaflets (PILs) and 40 antibiotic web resouces. Of these 85 PILs, 72 were taken from the National Medicines Regulator from six countries (3 TB high-incidence + 3 TB low-incidence countries), as listed in Fig. 1. Readability data was grouped into three categories, including (i) high-incidence countries (*n* = 33 information sources), (ii) low-incidence countries (*n* = 39 information sources) and (iii) web information (*n* = 53 information sources).

**Fig. 2:**
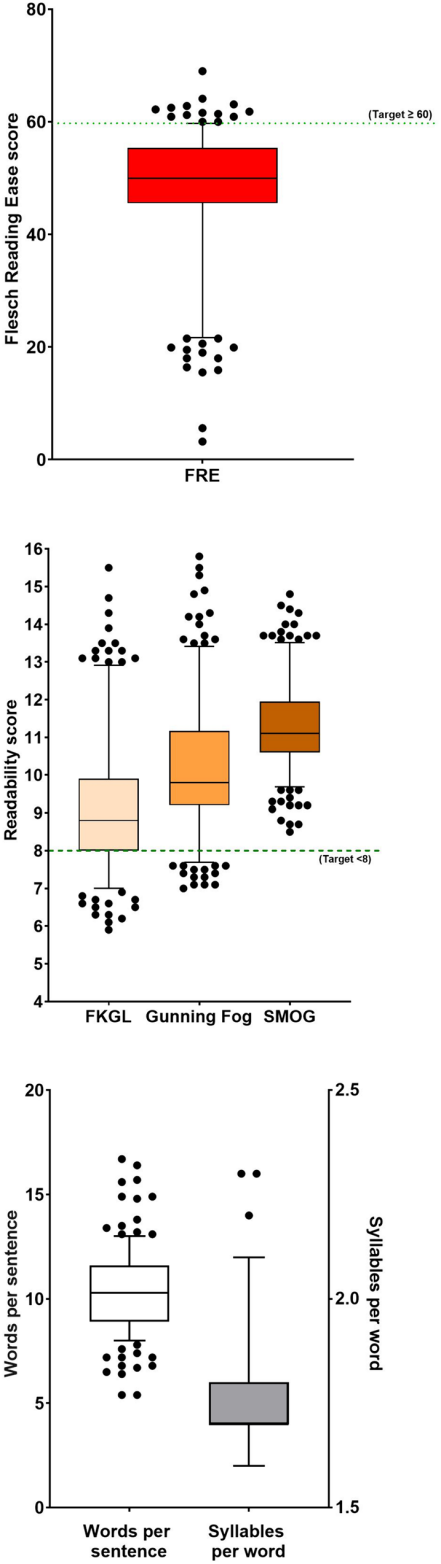
Box and whiskers plot comparing readability scores calculated on antibiotics (*n* = 10; bedaquiline, clofazimine, ethambutol, ethionamide, isoniazid, levofloxacin, linezolid, moxifloxacin, pretomanid, pyrazinamide) from patient-facing information from 125 sources [high TB-incidence countries (*n* = 33); lowTB-incidence countries (*n* = 39); web information (*n* = 53)]. **A** Flesch Reading Ease; **B** Flesch-Kincaid Grade Level; Gunning Fog Score; SMOG score; **C** Words per sentence; Syllables per word. Box represents 25th and 75th percentile and bar represents the median. Whiskers represent the 10th and 90th percentile and · represent outliers outside these percentile ranges. Statistical significance is shown, calculated using the Kruskal–Wallis (non-parametric) test with Dunn’s Adjusted p values. A p value of < 0.05 (5%) was considered as statistically significant. The dashed red line represents the target readability score. For the Flesch Reading Ease, this is $\ge$ 60. For the other readability scores, this is ≤ 8.

**Fig. 3:**
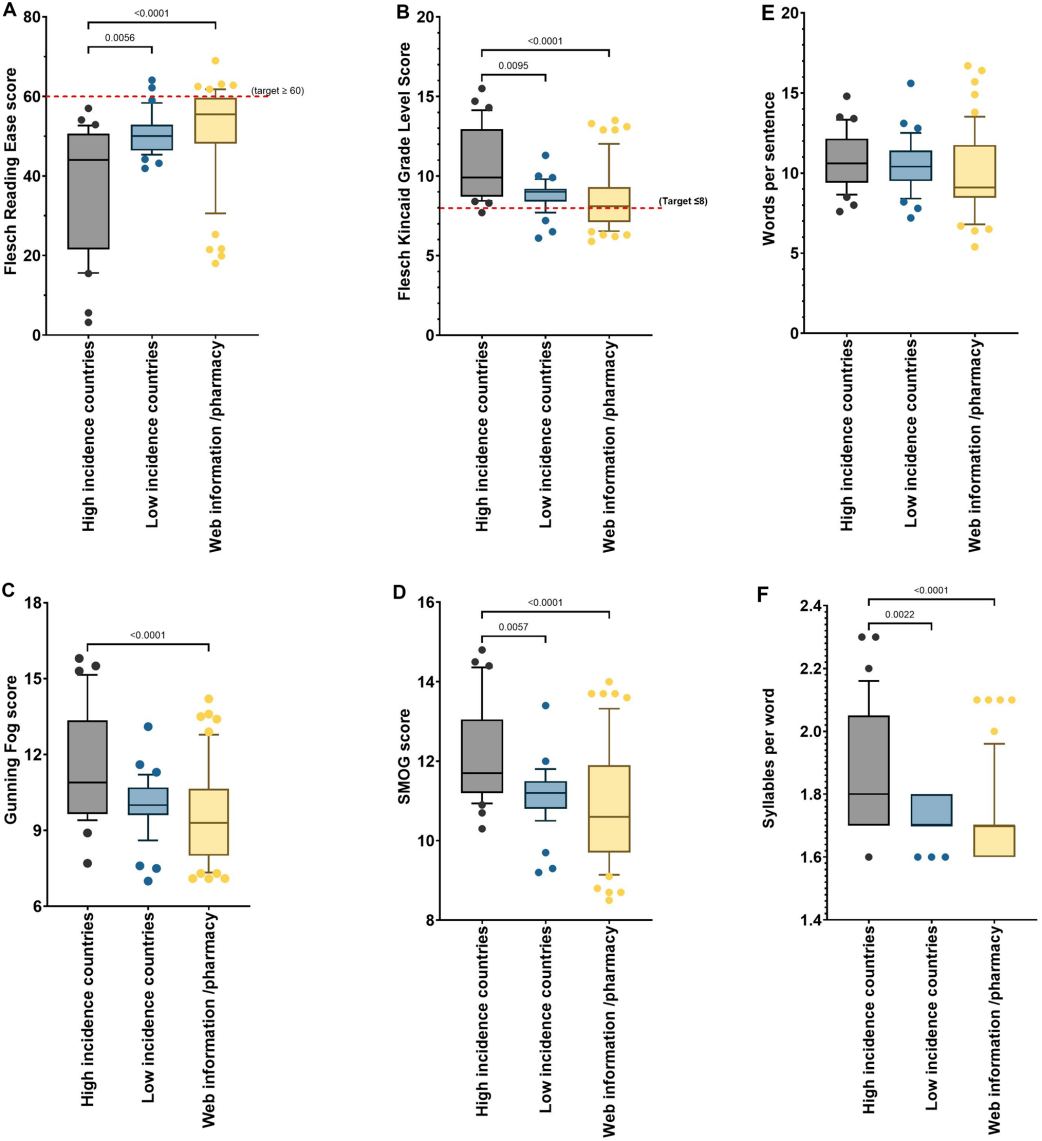
Box and whiskers plot comparing readability scores calculated on antibiotics (*n* = 10; bedaquiline, clofazimine, ethambutol, ethionamide, isoniazid, levofloxacin, linezolid, moxifloxacin, pretomanid, pyrazinamide) from patient-facing information from 125 sources comparing high TB-incidence countries (*n* = 33); lowTB-incidence countries (*n* = 39) and web information (*n* = 53)]. **A** Flesch Reading Ease; **B** Flesch-Kincaid Grade Level; **C** Gunning Fog Score; **D **SMOG score; **E** Words per sentence; **F** Syllables per word. Box represents 25th and 75th percentile and bar represents the median. Whiskers represent the 10th and 90th percentile and · represent outliers outside these percentile ranges. Statistical significance is shown, calculated using the Kruskal–Wallis (non-parametric) test with Dunn’s Adjusted p values. A p value of < 0.05 (5%) was considered as statistically significant. The dashed red line represents the target readability score. For the Flesch Reading Ease, this is $\ge$ 60. For the other readability scores, this is ≤ 8.

Readability scores for the Flesch Reading Ease, the Flesch-Kincaid Grade Level, the Gunning Fog score and the SMOG score, as well as for the two text metrics, words per sentence and syllables per word, are shown in Fig. 2A–C. Comparison of readability scores and text metrics amongst the three categories is shown (Fig. 3A–F). All data sets were found to be not normally distributed, therefore for comparison, the Kruskal–Wallis test and Dunn’s multiple comparisons test was employed to compare readability parameters between each patient-facing information source.

## Discussion

**Fig. 4:**
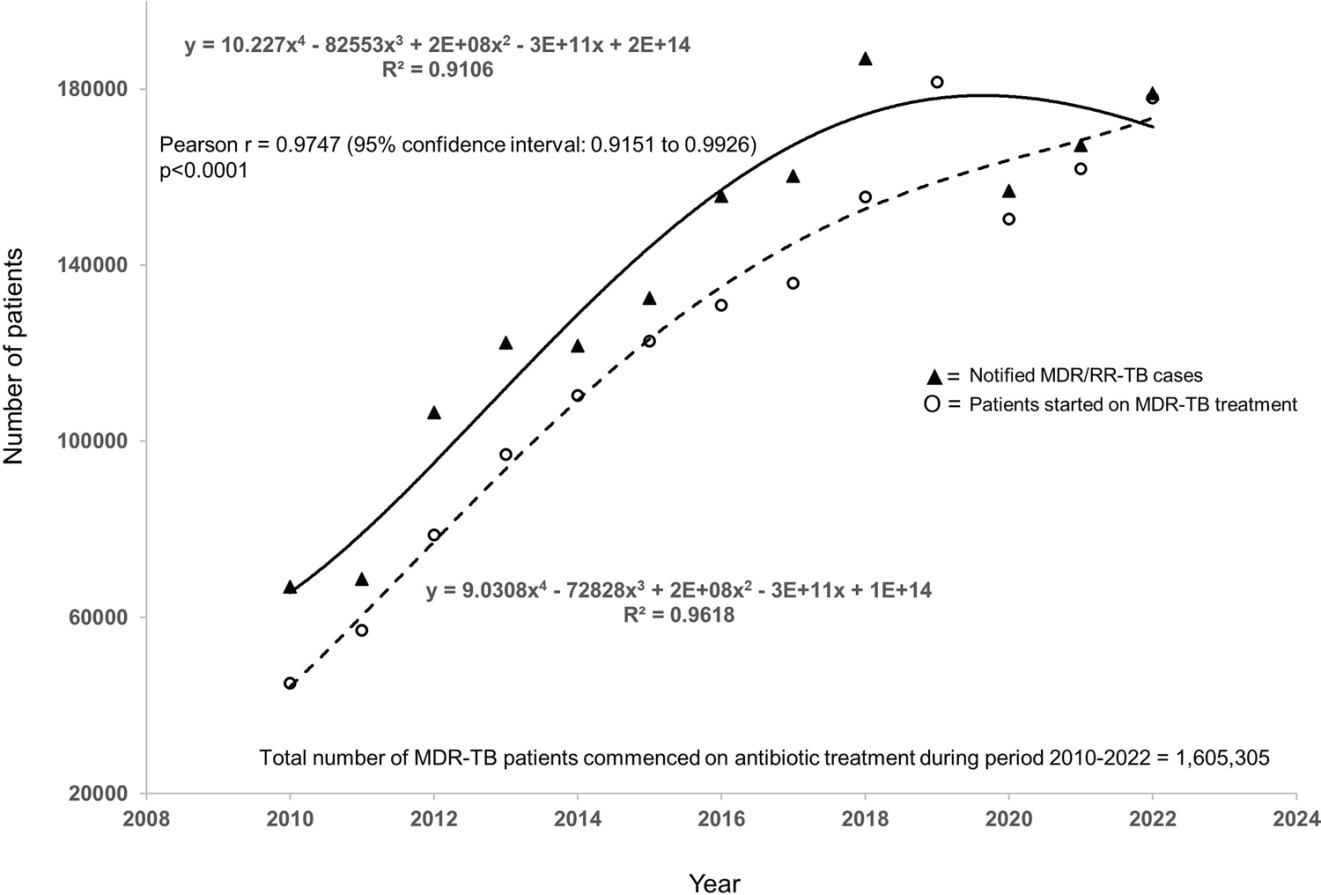
Correlation between notified MDR/RR-TB cases and patients started on MDR-TB treatment for the period 2010 and 2022. Source of data: https://www.who.int/data/gho/indicator-metadata-registry

All oral treatment regimes of drug-resistant TB have now become established TB pharmacotherapy, in line with WHO call for accelerated uptake of these all oral regimes [15]. Employing WHO data (https://www.who.int/teams/global-tuberculosis-programme/data), Fig. 4 shows the number of MDR-TB patients commenced on antibiotic treatment during the period 2010–2022. In a recent update, Gupta and colleagues showed that there has been consistent global growth in the use of shorter regimens in DR-TB treatment, with BPaLM reaching 126,792 patients, BPaL reaching 43,716 patients and the 9–11-month all-oral bedaquiline-based regimen reaching 13,119 patients by 2026 [16]. By 2026, it has been estimated that the longer all-oral regimen is projected to be used by 19,262 patients, and individualised treatment regimens by 15,344 patients [16].

This shift in antibiotic formulations from iv to orals has been largely driven by the discovery of newer effective oral antibiotics that have been shown to be safer the iv antibiotics [17, 18]. The lengthy duration of treating drug-resistant TB with ivs increases the toxicological burden associated with this administrative route, as examplified by iv kanamycin and capreomycin [19, 20]. Additionally, clinical trial data using all oral treatment combinations have yielded positive outcomes [21–23]. An open-label, phase 2–3, multicenter, randomized, controlled, noninferiority trial was conducted in Belarus, South Africa, and Uzbekistan to evaluate the efficacy and safety of three 24-week, all-oral regimens (bedaquiline, pretomanid, linezolid, and moxifloxacin), for the treatment of rifampin-resistant tuberculosis. Results showed that this all-oral regimen was non-inferior to the accepted standard-care treatment and it had a better safety profile [23, 24].

Parenteral administration of antibiotics in the treatment of DR-TB has also included the intramuscular (im) route, especially for kanamycin and capreomycin. This route employs deep im injection, with alternating injection sites [25]. This route of antibiotic administration has been problematic due to the injections being painful, especially for those with limited muscle mass, becoming intolerant to im injection due to pain at the injection site [26]. For this reason, im antibiotic administration has been cited as one of the worst aspects of DR-TB treatment [27].

The switch from using iv and im antibiotics to exclusively oral antibiotics creates new challenges, particularly with treatment adherence and compliance. Employment of iv antibiotics involve allied healthcare professionals assisting with their administration to the patient, whereas with oral antibiotics, administering of these orals is largely goverened by the patient themselves. Therefore, antibiotic treatment adherence may be a bigger problem with solely oral regimes than with those regimes involving an iv antibiotic and an issue that requires careful reflection. A recent systematic review by Pradipta and colleagues of 14 studies, including 10 active TB and four latent TB studies showed that directly observed treatment (DOT) by trained community workers, short messaging service combined with education, counselling, monthly TB vouchers, drug box reminders and combinations of those were effective [6]. Previously, in a high DR-TB burden setting in Khayelitsha, Cape Town, South Africa, Mohr and colleagues describe their development of a patient-centric approach to DR-TB treatment that was integrated into existing TB and HIV primary care programmes [28]. Their model involved structured and standardised adherence support sessions that were developed into a DR-TB counselling toolkit, focussing on DR-TB treatment literacy, adherence strategies to encourage retention in care and provision of support throughout the patient’s long treatment journey [28].

The WHO describes the monitoring schedule for patients receiving the 9-month all oral MDR/RR-TB regimen, which includes the component “*Treatment literacy and adherence counselling*”, with inputs from this component at baseline, and at 0–2 months, at every healthcare worker interaction and then, as necessary for the following seven months [4]. However, there is no further discussion on what interventions or monitoring should entail with “*Treatment literacy and adherence counselling*”. Literacy plays an important role in the understanding of tuberculosis [29]. Developing resources for TB patients to support treatment literacy of the new WHO short 6-and 9 month all oral treatment regimes would be prudent, in order to help patients better understand their antibiotic medications, as well as dosing and treatment durations, in an attempt to maximise treatment adherence.

The patient information leaflet (PIL) is an important source of information for the patient, which accompanies presciption medicine and which is intended to help the patient understand key aspects of the medication for their treatment. PILs, which accompany medication, including antibiotics, have been shown to have a positive impact on medication adherence [31]. In this study by Al Jeraisy and colleagues in Saudi Arabia involving 1138 adult individuals, the practice of patients reading the PILs positively impacted their medication adherence (64.9%), whilst 8.8% of respondents indicated that reading the PIL negatively impacted on their adherence, due to concerns surrounding the medicines’ side effects and complications. Further data from India showed that PILs significantly improved patients knowledge about their medication and improved compliance at home [32]. Unfortunately there are no reports of audits checking whether or not every medicine is accompanied by a PIL within the packaging. In Western countries, the supply of the PIL is mandatory, accompanying each precription medication. Where repeat medication is supplied on a daily basis by a healthcare provider, the DR-TB patient should have initial access to the PIL for each medicine taken and be asked regularly if they would like an update on any information that the PIL describes.

To our knowledge, the current study is the first to conduct an assessment of the readability of PILs of antibiotics employed in the treatment of drug-resistant TB, according to the WHO guidelines [4]. In this study, we employed quantitaive measurement of words, sentences and syllables, as defined by readability formulae, including Flesch Reading Ease, Flesch Kincaid Grade Level, Gunning Fog and SMOG scores (Supplementary Table 1). Readability has now become a commonly employed tool to help healthcare professionals prepare patient-facing materials and resources, supported by a growing evidence-base of published literature, where currently there are approximately 500 publications cited in PubMed per year, devoted to its study and application within clinical medicine, particularly its value with patient-facing information and material resources. To date, an advanced PubMed search of the title terms “readability” and “antibiotic” returns one sole publication from our group, [33] describing its value amongst patients with cystic fibrosis, thereby demonstrating the novelty and opportunity of the application of such an approach to promote antibiotic usage awareness and treatment literacy amongst TB patients, receiving short all oral antibiotic regimes [33].

The design of our study involved the analyses of readability of PILs of the 10-listed oral antibiotics, defined in the WHO BPaL, BPaLM and BPaLC antibiotic regimens [4]. PILs information was sourced from three groups, namely (i) high TB incidence countries, including Rwanda (TB rate per 100,000 population (2022) (Data source: WHO available at https://worldhealthorg.shinyapps.io/tb_profiles/?_inputs_&entity_type=%22country%22&iso2=%22RW%22&lan=%22EN%22 [56/100,000), Malaysia (113/100,000) and South Africa (468/100,000), (ii) low incidence countries, including UK (7.6/100,000), Ireland (4.5/100,000) and Malta (13/100,000), as well as (iii) TB drug information web resources. All PILs from (i) and (ii) were sourced from websites of the medicines regulator of each respective country. Our first observation was that PILs information was difficult to source online from the majority of medicine regulators globally. More regulators listed the Summary of Product Characteristics (SPC) of these antibiotics, however these are not designed to be patient-facing, but rather healthcare professional-facing.

From examination and comparison of the readability and text metrics results of this study, the overall readability and text metric scores from all sources combined did not meet the readability reference targets of ≥ 60, for the Flesch Reading Ease score and ≤ 8, for the Flesch Kincaid Grade Level (Fig. 2A–C). Recommendations for suitable readability levels can vary between institutions, with the American Medical Association recommending that all patient-facing material be written at a sixth grade level (11 years old) [14]. Conversely, the Centers for Disease Control and Prevention (CDC) recommends that patient-facing information does not surpass an eighth grade reading level (13 years old) [14].

Only 11/125 (8.8%) of TB antibiotc information sources met this target and 12/125 (9.6%) reaching the target level for the Flesch Kincaid Grade Level. This indicates TB antibiotic information is not considered to be written adequately for the public and are thus too difficult for the general public to read. When the information sources within these three categories were compared to each other, the most readable of all the information sourced, was that from reliable internet web resources, including the US government’s National Library of Medicine, MedlinePlus, as well as the electronic medicines compendium (EMC) and Drugs.com. PILs from high TB incidence countries consistently had the lowest readability scores, when compared to low incidence countries and web resources (Flesch Reading Ease; *p* = 0.0056 and *p* < 0.0001, respectively) (Fig. 3A). There was no statistical differences (*p* > 0.05) in readability or text metrics scores between low incidence countries and web resources. Poor readability scores were associated with higher words per sentence and higher syllables per word text metrics.

**Fig. 5:**
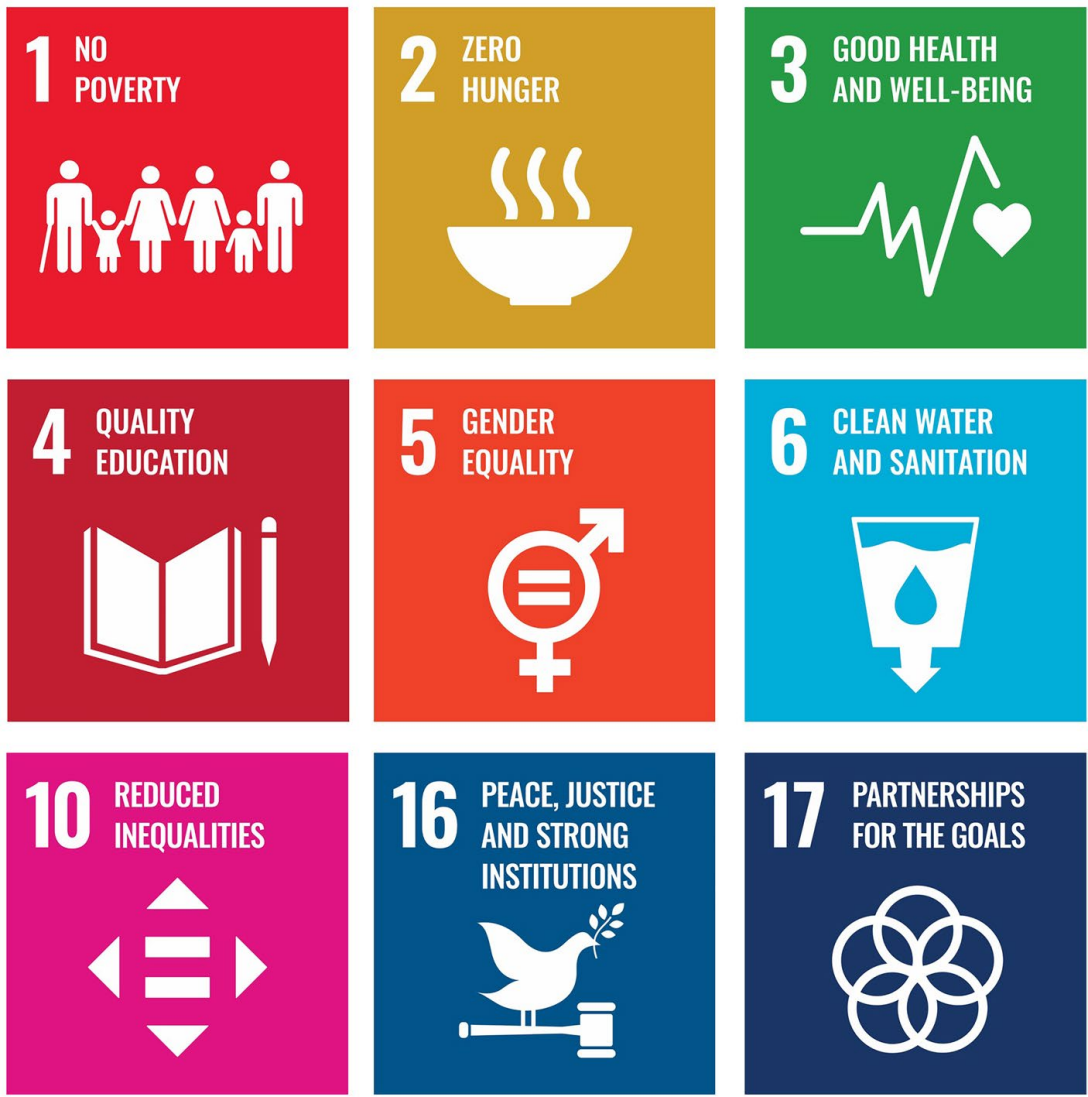
Alignment of identified and relevant UN Sustainable Development Goals (SDGs) associated with poor readability of TB antibiotic treatment information

It is concerning to note that the poorest readability scores were PILs from high incidence countries. PILs are essentially designed to inform the patient with important information regarding their medication, to allow patients the choice and enable them to make knowledgeable and responsible decisions with regard to their medications [34]. Therefore, it is important that PILs are easily accessible by inclusion in community-dispensed medicines and which are easily read and their value promoted to patients and service users, by the respiratory team and pharmacist treating the TB patient. The issues of poor readability of TB antibiotic PILs in the context of treating drug-resistant tuberculosis, as identified in the current study, is in itself a microcosm of a multitude of interwoven societal problems. We have reflected on these issues and have subsequently aligned those relevant UN Sustainable Development Goals to the issues of poor readability of TB antibiotic PILs, as shown in Fig. 5.

### Limitations and Future Work

The study presented here has several limitations. Firstly, the PILs collected and analysed were limited to the English language only, thereby making it of most value to the study of TB treatment and adherence in English-speaking countries. All non-English patient information sources were excluded from this study. This was due to the online readability tool (*Readable*), employed in this study, being best suited for scoring texts using the English alphabet, as it is not able to assess readability of texts written with alternative characters, such as Arabic, Chinese and Japanese. Another limitation was the lack of availability of PILs from the majority of countries, particularly high incidence countries, which would have reflected more robust representation of readability of PILs from high incidence countries. Where English is not the first language and where countries have a high rate of illiteracy, governments, NGOs and public health agencies should consider an alternative to the written PIL and adopt alternative media, such as video, animation or podcast, to allow high quality antibiotic information to be disemminated, as an alternative to the traditional PIL, thereby ensuring the same quality of public health messaging to maximise antibiotic treatment adherence. Additionally, countries should ensure that patients have independent and easy access to a source of high quality information on TB antibiotics, in an understandible and comprehensible format, matching the literacy and health literacy baseline values of that country, so that lack of knowledge about antibiotics is not allowed to translate into poor antibiotic adherence and onwards to poor clinical outcomes.

In conclusion, readability of PILs of the 10 antibiotics listed in WHO short, all-oral treatment regimens is poor, not reaching readability reference standards. Such poor readability could be reflected in poor understandibility, leading to non-compliances in patient-centred TB treatment regimens, cumulating in poor disease outcomes. To date, readability of antibiotic PILs has not been scrutinised, nor has it been considered as an integral intervention of TB treatment and patient health literacy. Authors of antibiotic PILs and other TB antibiotic information should consider the adoption of readability calculators when preparing medication information for TB patients, to check the readability of their work, so that the final material is within recommended readability reference parameters, to support the health literacy and treatment adherence of their readers, as well as maximising the value of the Patient Information Leaflet in independent, reliable and trusted TB information dessimination to TB patients globally.

## Supplementary Information

Below is the link to the electronic supplementary material.

Supplementary file1 (PDF 371 KB)

Supplementary file2 (PDF 287 KB)

## Data Availability

All data supporting the findings of this report are freely available in the public domain for access by readers. No unique datasets were generated in this study.

## References

[1] Kim PS, Swaminathan S (2021) Ending TB: the world’s oldest pandemic. J Int AIDS Soc 24:e25698. 10.1002/jia2.25698 33754449 10.1002/jia2.25698 PMC7985566

[2] World Health Organization (WHO). Global tuberculosis report 2023. Geneva. Licence: CC BY-NC-SA 3.0 IGO. Available at https://www.who.int/teams/global-tuberculosis-programme/tb-reports [Last accessed 10 July 2024].

[3] Dheda K, Mirzayev F, Cirillo DM et al (2024) Multidrug-resistant tuberculosis. Nat Rev Dis Primers 10:22. 10.1038/s41572-024-00504-2 38523140 10.1038/s41572-024-00504-2 PMC13335523

[4] World Health Organization. WHO operational handbook on tuberculosis. Module 4: treatment - drug-resistant tuberculosis treatment, 2022 update. Available at https://www.who.int/publications/i/item/9789240065116 [Last accessed 09 May 2024].

[5] Mohr E, Daniels J, Beko B et al (2017) DOT or SAT for rifampicin-resistant tuberculosis? A non-randomized comparison in a high HIV-prevalence setting. PLoS ONE 12:e0178054. 10.1371/journal.pone.0178054 28542441 10.1371/journal.pone.0178054 PMC5436852

[6] Pradipta IS, Houtsma D, van Boven JFM et al (2020) Interventions to improve medication adherence in tuberculosis patients: a systematic review of randomized controlled studies. NPJ Prim Care Respir Med 30:21. 10.1038/s41533-020-0179-x 32393736 10.1038/s41533-020-0179-x PMC7214451

[7] Yamamoto Y, Kadota J, Watanabe A et al (2012) Compliance with oral antibiotic regimens and associated factors in Japan: compliance survey of multiple oral antibiotics (COSMOS). Scand J Infect Dis 44:93–99. 10.3109/00365548.2011.619998 22017766 10.3109/00365548.2011.619998

[8] Chimeh RA, Gafar F, Pradipta IS et al (2020) Clinical and economic impact of medication non-adherence in drug-susceptible tuberculosis: a systematic review. Int J Tuberc Lung Dis 24:811–819. 10.5588/ijtld.19.0754 32912386 10.5588/ijtld.19.0754

[9] Palomino JC, Martin A (2014) Drug resistance mechanisms in *Mycobacterium tuberculosis*. Antibiotics 3:317–340. 10.3390/antibiotics3030317 27025748 10.3390/antibiotics3030317 PMC4790366

[10] Munsour EE, Awaisu A, Hassali MAA et al (2017) Readability and comprehensibility of patient information leaflets for antidiabetic medications in Qatar. J Pharm Technol 33:128–136. 10.1177/8755122517706978 34860991 10.1177/8755122517706978 PMC5998532

[11] Badarudeen S, Sabharwal S (2010) Assessing readability of patient education materials: current role in orthopaedics. Clin Orthop Relat Res 468:2572–2580. 10.1007/s11999-010-1380-y 20496023 10.1007/s11999-010-1380-y PMC3049622

[12] Meleo-Erwin Z, Basch C, Fera J et al (2019) Readability of online patient-based information on bariatric surgery. Health Promot Perspect 9:156–160. 10.15171/hpp.2019.22 31249804 10.15171/hpp.2019.22 PMC6588814

[13] Patel PA, Gopali R, Reddy A et al (2022) The readability of ophthalmological patient education materials provided by major academic hospitals. Semin Ophthalmol 37:71–76. 10.1080/08820538.2021.1915341 33852375 10.1080/08820538.2021.1915341

[14] McGrath L, Millar BC, Moore JE (2022) Using plain language to communicate with clinical trials participants: comparison of readability calculators. Contemp Clin Trials 123:106995. 10.1016/j.cct.2022.106995 36347454 10.1016/j.cct.2022.106995

[15] Holger DJ, Althubyani A, Morrisette T et al (2024) Updates in pulmonary drug-resistant tuberculosis pharmacotherapy: a focus on BPaL and BPaLM. Pharmacotherapy 44:268–282. 10.1002/phar.2909 38270468 10.1002/phar.2909

[16] Gupta A, Juneja S, Babawale V et al (2024) Global adoption of 6-month drug-resistant TB regimens: projected uptake by 2026. PLoS ONE 19:e0296448. 10.1371/journal.pone.0296448 38180980 10.1371/journal.pone.0296448 PMC10769048

[17] World Health Organization. WHO Consolidated Guidelines on Tuberculosis. Module 4: Treatment—Drug-Resistant Tuberculosis Treatment, 2022. Available at https://www.who.int/publications/i/item/9789240048126 [Last accessed 09 May 2024]. 32603040

[18] Nahid P, Mase SR, Migliori GB et al (2019) Treatment of drug-resistant tuberculosis. An official ATS/CDC/ERS/IDSA clinical practice guideline. Am J Respir Crit Care Med 200:e93–e142. 10.1164/rccm.201909-1874ST 31729908 10.1164/rccm.201909-1874ST PMC6857485

[19] Genestet C, Ader F, Pichat C et al (2017) Assessing the combined antibacterial effect of isoniazid and rifampin on four *Mycobacterium tuberculosis* strains using in vitro experiments and response-surface modeling. Antimicrob Agents Chemother 62:e01413-e1417. 10.1128/AAC.01413-17 29061753 10.1128/AAC.01413-17 PMC5740320

[20] Cegielski JP, Chan PC, Lan Z et al (2021) Aminoglycosides and capreomycin in the treatment of multidrug-resistant tuberculosis: individual patient data meta-analysis of 12,030 patients from 25 countries, 2009–2016. Clin Infect Dis 73:e3929–e3936. 10.1093/cid/ciaa621 33124668 10.1093/cid/ciaa621 PMC8653626

[21] Conradie F, Diacon AH, Ngubane N et al (2020) Treatment of highly drug-resistant pulmonary tuberculosis. New Engl J Med 382:893–902. 10.1056/NEJMoa1901814 32130813 10.1056/NEJMoa1901814 PMC6955640

[22] Conradie F, Bagdasaryan TR, Borisov S et al (2022) Bedaquiline-pretomanid-linezolid regimens for drug-resistant tuberculosis. New Engl J Med 387:810–823. 10.1056/NEJMoa2119430 36053506 10.1056/NEJMoa2119430 PMC9490302

[23] Nyang’wa BT, Berry C, Kazounis E et al (2022) 24-week, all-oral regimen for rifampin-resistant tuberculosis. New Engl J Med 387:2331–2343. 10.1056/NEJMoa2117166 36546625 10.1056/NEJMoa2117166

[24] Nyang’wa BT, Berry C, Kazounis E et al (2024) Short oral regimens for pulmonary rifampicin-resistant tuberculosis (TB-PRACTECAL): an open-label, randomised, controlled, phase 2B–3, multi-arm, multicentre, non-inferiority trial. Lancet Respir Med 12:117–128. 10.1016/S2213-2600(23)00389-2 37980911 10.1016/S2213-2600(23)00389-2

[25] Zhu M, Burman WJ, Jaresko GS et al (2001) Population pharmacokinetics of intravenous and intramuscular streptomycin in patients with tuberculosis. Pharmacotherapy 21:1037–1045. 10.1592/phco.21.13.1037.34625 11560193 10.1592/phco.21.13.1037.34625

[26] Garcia-Prats AJ, Rose PC, Draper HR et al (2018) Effect of coadministration of lidocaine on the pain and pharmacokinetics of intramuscular amikacin in children with multidrug-resistant tuberculosis: a randomized crossover trial. Pediatr Infect Dis J 37:1199–1203. 10.1097/INF.0000000000001983 29561515 10.1097/INF.0000000000001983 PMC6138584

[27] Isaakidis P, Rangan S, Pradhan A et al (2013) ‘I cry every day’: experiences of patients co-infected with HIV and multidrug-resistant tuberculosis. Trop Med Int Health 18:1128–1133. 10.1111/tmi.12146 23837468 10.1111/tmi.12146

[28] Mohr E, Hughes J, Snyman L et al (2015) Patient support interventions to improve adherence to drug-resistant tuberculosis treatment: a counselling toolkit. S Afr Med J 105:631–634. 10.7196/samj.10093 26449704 10.7196/samj.10093

[29] Chauhan A, Parmar M, Dash GC et al (2024) Health literacy and tuberculosis control: systematic review and meta-analysis. Bull World Health Organ 102:421 38812804 10.2471/BLT.23.290396 PMC11132163

[30] Sveinbjornsdottir GM, Kamowa D, Katundu PN et al (2024) Compliance and illiteracy when treating tuberculosis. Int Health 16:126–128. 10.1093/inthealth/ihad077 37655852 10.1093/inthealth/ihad077 PMC10759289

[31] Al Jeraisy M, Alshammari H, Albassam M et al (2023) Utility of patient information leaflet and perceived impact of its use on medication adherence. BMC Public Health 23:488. 10.1186/s12889-023-15346-y 36918823 10.1186/s12889-023-15346-y PMC10012310

[32] Gupta U, Sharma S, Sheth PD et al (2005) Improving medicine usage through patient information leaflets in India. Trop Doct 35:164–166. 10.1258/0049475054620644 16105344 10.1258/0049475054620644

[33] Tang KWK, Millar BC, Moore JE (2023) Improving health literacy of antibiotic use in people with cystic fibrosis (CF)-comparison of the readability of patient information leaflets (PILs) from the EU, USA and UK of 23 CF-related antibiotics used in the treatment of CF respiratory infections. JAC Antimicrob Resist. 10.1093/jacamr/dlad129 38046567 10.1093/jacamr/dlad129 PMC10691746

[34] Algabbani AM, Alzahrani SA, Almomen SM et al (2022) Readability of information imprinted in patient information leaflets (PILs) in Saudi Arabia: the case of antihypertensive medications. Explor Res Clin Soc Pharm 8:100179. 10.1016/j.rcsop.2022.100179 36177271 10.1016/j.rcsop.2022.100179 PMC9513263

