# Supplementary material for: Readability of Patient-Facing Information of Antibiotics Used in the WHO Short 6-Month and 9-Month All Oral Treatment for Drug-Resistant Tuberculosis
Source: Lung. 2024 Jul 26;202(5):741–51. doi: 10.1007/s00408-024-00732-z (PMC11427546; doi:10.1007/s00408-024-00732-z)
Supplement: Supplementary file 1 — Supplementary file1 (PDF 371 KB) [file 408_2024_732_MOESM1_ESM.pdf]

Supplementary File 1: Estimated number of people who developed MDR/RR-TB (incident cases) in 2022, for countries with at least 1000 incident cases*

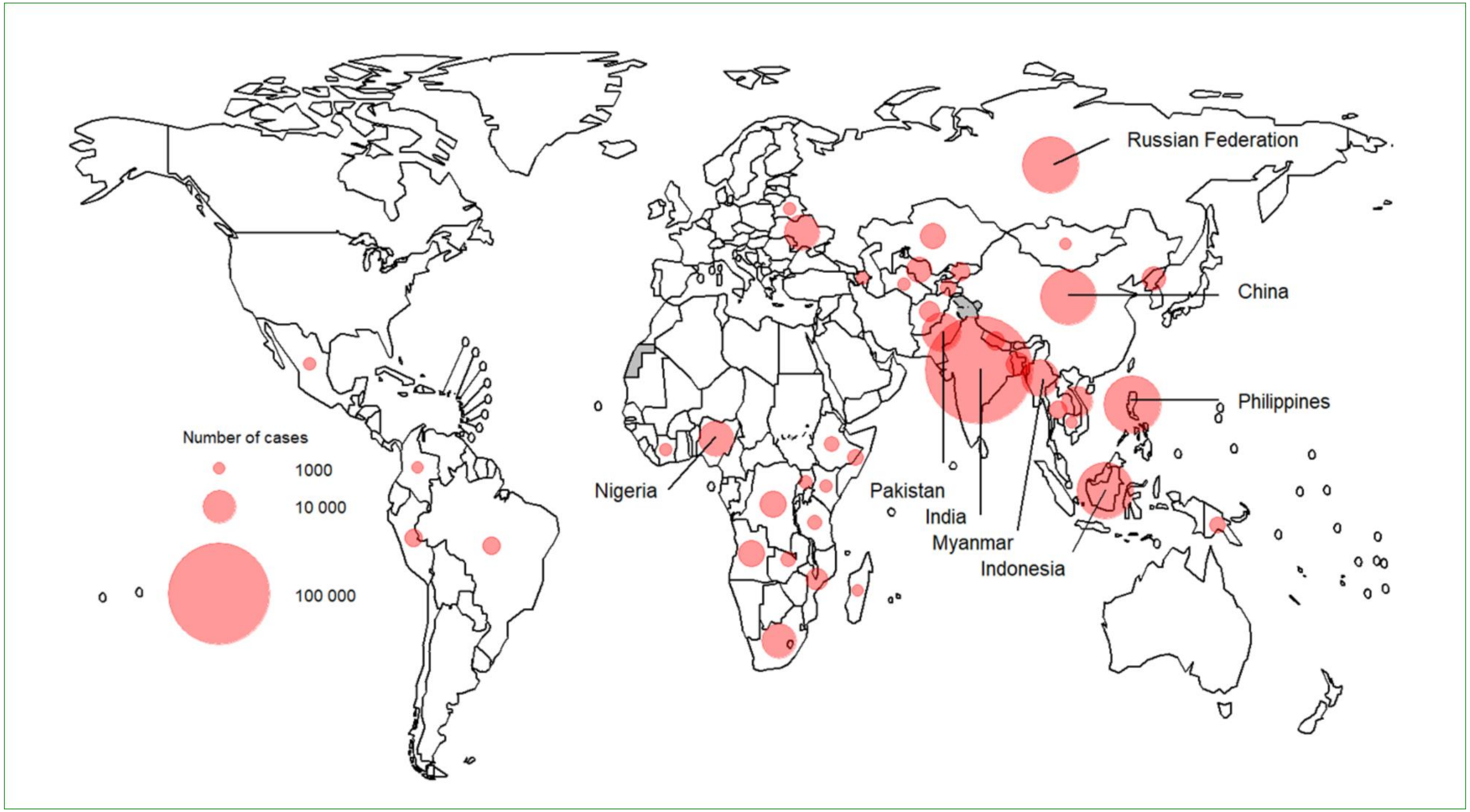

*Eight countries ranked in descending order of their total number of MDR/RR-TB incident cases in 2022 are India, the Philippines, the Russian Federation, Indonesia, China, Pakistan, Myanmar and Nigeria. The countries with the largest share of the global number of people estimated to have developed MDR/RR-TB (incident cases) in 2022 were India (27% of global cases), the Philippines (7.5% of global cases) and the Russian Federation (7.5% of global cases).

Reproduced from: *1.3 Drug-resistant TB, Global Tuberculosis Report 2023. World Health Organisation*. Available at https://www.who.int/teams/global-tuberculosis-programme/tb-reports/global-tuberculosis-report-2023/tb-disease-burden/1-3-drug-resistant-tb#fig--1-3-5 under a Creative Commons Attribution-NonCommercial-ShareAlike 3.0 Intergovernmental Organization (CC BY-NC-SA 3.0 IGO) licence (https://creativecommons.org/licenses/by-nc-sa/3.0/igo/). No changes were made.
