# Supplementary material for: Readability of Patient-Facing Information of Antibiotics Used in the WHO Short 6-Month and 9-Month All Oral Treatment for Drug-Resistant Tuberculosis
Source: Lung. 2024 Jul 26;202(5):741–51. doi: 10.1007/s00408-024-00732-z (PMC11427546; doi:10.1007/s00408-024-00732-z)
Supplement: Supplementary file 2 — Supplementary file2 (PDF 287 KB) [file 408_2024_732_MOESM2_ESM.pdf]

Supplementary Table 1: Summary table of readability formulae used in this study and their associated target scores for the general public.

Readability Index Name	Grade level	Formula	Target Score
Flesch-Kincaid Grade Level Score (FKGL)*	8	0.39 (total words/total sentences) + 11.8 (total syllables/total words) – 15.59	A text aimed at the general audience should have a grade level of 8 or lower. At Grade Level 8, 85% of the general population will be able to read and comprehend the text
The Gunning Fog Index (GF)*	7-8	0.4 x [(words ÷ sentences) + 100 x (complex words ÷ total words)]	Grade level 8 or below
Simple Measure of Gobbledygook (SMOG)*	8	3 + square root √ [number of polysyllabic words x (30 ÷ number of sentences)]	Grade level 8 or below
Flesch Reading Ease Score (FRE)**		206.835 – 1.015 x (total words ÷ total sentences) – 84.6 x (total syllables ÷ total words)	A target score of 60 or above.

*score decreases with ease of reading. ** scores increases with ease of reading Readability formulae are taken from Readable.com
